# Comment on Hawez et al. Endometrial Intraepithelial Neoplasia, Concurrent Endometrial Cancer and Risk for Pelvic Sentinel Node Metastases. *Cancers* 2024, *16*, 4215

**DOI:** 10.3390/cancers17081315

**Published:** 2025-04-14

**Authors:** Fatih Gücer, Jan Dünnebacke

**Affiliations:** Department of Obstetrics and Gynecology, Catholic Clinics of Koblenz-Montabaur, Marienhof Koblenz, Rudolf Virchow Str. 7–9, 56073 Koblenz, Germany; j.duennebacke@bbtgruppe.de

We read with interest the recent paper by Hawez et al. titled “Endometrial Intraepithelial Neoplasia, Concurrent Endometrial Cancer and Risk for Pelvic Sentinel Node Metastases”, published in the journal *Cancers* [1]. The data presented in the article are of clinical importance and make a significant contribution to the literature.

In this prospective study, the authors shared their clinical experience from a tertiary referral center in Sweden. The study included 98 consecutive patients diagnosed with endometrial intraepithelial neoplasia, all of whom underwent sentinel lymph node dissection followed by robotic total hysterectomy with bilateral salpingo-oophorectomy as their definitive surgical treatment approach. All patients were staged according to their surgical pathological findings, using FIGO 2019 staging.

The authors stated that 47% of patients with EIN endometrial cancer were diagnosed upon final histopathology. Six out of forty-six patients with concomitant endometrial cancer (13%) had sentinel lymph node involvement in different manners and were staged as FIGO stage IIIC1 (n = 4) and FIGO stage IIIC2 (n = 2). The summary of metastatic cases was presented in Table 1 in the original paper. Notably, there were no cases of macrometastases in the sentinel lymph nodes. However, in five out of six patients with malignant cells in the sentinel lymph nodes, the metastatic dissemination was present only in the form of isolated tumor cells (ITCs).

Based on the presence of malignant cells in the sentinel lymph nodes, the authors reported the risk of malignant dissemination in the EIN group as 6.3%. Although the study period spanned from July 2019 to May 2024, the authors chose to use the older version of the FIGO staging system from 2019. On the other hand, the latest FIGO staging system from 2023 distinguishes patients with ITCs from those with micro- or macrometastases and specifies that ITCs should be noted separately [2]. Contrary to the authors’ classification in Table 1 in the original article, according to the current FIGO classification, the presence of ITCs alone does not indicate upstaging to FIGO stage IIIC1 or IIIC2 if the disease is otherwise confined to the uterus. Therefore, we were curious about the rationale for staging these patients with ITCs as IIIC1 or IIIC2.

Since the presence of ITCs in patients with endometrial cancer does not affect the FIGO staging or does not indicate the need for additional treatment, it is important to have a clear statement from professional societies regarding whether cases with ITCs in the lymph nodes should be included when investigating, like the present study, the incidence of the malignant involvement of sentinel lymph nodes in EIN. If not, the “true” and “consequent” rate of malignant involvement in sentinel lymph nodes for patients with EIN and concurrent endometrial cancer would be calculated without considering the presence of ITCs. In this case, the rate reported by Hawez et al. would be 1.02% (instead of 6.3%). This issue is crucial for providing accurate and thorough counseling to patients with EIN regarding sentinel lymph node biopsies.
